# Maize Aldehyde Decarbonylase 1 Gene (*ZmCER1*) Positively Regulates Salt and Drought Tolerance by Improving Wax Synthesis and Reactive Oxygen Species Detoxification

**DOI:** 10.3390/cimb48050509

**Published:** 2026-05-14

**Authors:** Yaqing Yang, Mingzi Shi, Yaxin Liu, Xiaomei Gao, Hui Li, Laming Pei

**Affiliations:** 1 School of Biological Science and Technology, University of Jinan, Jinan 250022, China; yaqingyang2022@163.com (Y.Y.);; 2 Shandong Engineering Research Center of Key Technologies for High-Value and High-Efffciency Full Industry Chain of Lonicera Japonica, Linyi 273399, China

**Keywords:** maize epicuticular waxes, ZmCER1, salt stress, drought stress, antioxidant enzymes, ZmPEX14

## Abstract

Maize (*Zea mays* L.) is a vital global crop whose productivity is severely threatened by abiotic stresses. Epicuticular waxes provide a hydrophobic barrier that protects land plants from environmental stresses. However, the role of key wax biosynthetic enzymes, such as aldehyde decarbonylase CER1, in maize stress adaptation remains unclear. In this study, we performed a functional characterization of *ZmCER1* in maize. Our results show that the overexpression of *ZmCER1* in both Arabidopsis and maize substantially improved tolerance to these abiotic stresses. Under stress conditions, the transgenic plants displayed better growth performance, elevated activities of antioxidant enzymes, and reduced levels of oxidative damage markers. Additionally, the alkane content—especially that of C29 and C31—was significantly increased in the *ZmCER1OE* lines. Through a yeast two-hybrid screening (Y2H screening), we identified the peroxisomal membrane protein ZmPEX14 as an interacting partner of ZmCER1, and the interaction was further confirmed by luciferase complementation (LUC) and bimolecular fluorescence complementation (BiFC) assays. We propose a model wherein *ZmCER1* enhances stress tolerance not only by reinforcing the cuticular wax barrier but also by potentially regulating reactive oxygen species (ROS) detoxification via association with ZmPEX14. Collectively, our findings establish *ZmCER1* as a key regulator of abiotic stress tolerance in maize and a promising candidate for the molecular breeding of stress-resilient crops.

## 1. Introduction

Maize (*Zea mays* L.) stands as a foundation of global agriculture and food security, serving as a primary staple food for over 4.5 billion people, a critical material for livestock production, and a versatile raw material for industries ranging from biofuel to bioplastics [1]. However, maize growth and grain yield are highly susceptible to various abiotic stresses [2]. Drought-induced water scarcity affects nearly all the climatic zones worldwide. Such conditions can persist for extended periods, impeding maize growth and resulting in substantial yield reductions [3,4]. Soil salinization has emerged as another major global challenge, limiting maize yield potential [5,6]. A common consequence of both drought and salinity stress is the disruption of cellular homeostasis, leading to the accelerated production of reactive oxygen species (ROS) such as hydrogen peroxide (H_2_O_2_), superoxide radicals (O^2−^), and hydroxyl radicals (OH•) [7]. At high concentrations, ROS cause oxidative damage to vital cellular components, including lipids, proteins, and DNA. Malondialdehyde (MDA), a byproduct generated during the peroxidation of polyunsaturated fatty acids in cell membranes, serves as a key indicator of oxidative damage [8]. To counteract this oxidative damage, plants have evolved a sophisticated antioxidant defense system comprising non-enzymatic antioxidants and key enzymes such as superoxide dismutase (SOD), peroxidase (POD) and catalase (CAT), which work in concert to scavenge ROS and maintain redox balance [9]. A plant’s capacity to rapidly and efficiently activate its intrinsic antioxidant defense system is strongly correlated with its tolerance to various abiotic stresses. Another critical line of defense, particularly against drought, is the plant cuticle—a hydrophobic layer covering the aerial surfaces of plants composed of cutin and epicuticular waxes. Serving as a critical protective barrier, the cuticular wax layer effectively restricts non-stomatal water loss, mitigates UV radiation-induced harm, and blocks the invasion of phytopathogens [10,11,12,13]. The cuticle mainly consists of two types of lipophilic material, cutin and cuticular wax. Cutin is the major structural component of the cuticle and is composed of hydroxy and epoxy C16 and C18 fatty acid monomers, as well as glycerol [14]. Cuticular waxes consist of complex mixtures of hydrophobic compounds, mostly very-long-chain fatty acids (VLCFAs) with more than 20 carbon atoms and their derivatives, including primary and secondary alcohols, aldehydes, alkanes, ketones, and wax esters [10,11,15]. The wax components are produced according to two different pathways: (1) the alcohol-forming pathway, which generates very-long-chain (VLC) primary alcohols and wax esters; and (2) the alkane-forming pathway, responsible for the production of VLC alkanes and their derivatives [16]. In addition, alkanes are the prominent components of wax in the stem and leaf epidermis of higher plants, playing an extremely important role in controlling epidermal permeability [17,18]. Substantial progress has been made in recent decades in identifying the genes involved in alkane biosynthesis. Aldehyde decarbonylase, a member of the fatty acid hydroxylase (FAH) superfamily, serves as a key enzyme in the synthesis of very-long-chain alkanes (VLCAs) and contributes to plant responses to abiotic stresses [17,19,20]. In Arabidopsis, *ECERIFERUM 1* (CER1) encodes an aldehyde decarbonylase that participates in the alkane-forming pathway and modulates stress tolerance [17,21,22]. Subsequent studies have established that Arabidopsis *CER1* and *CER3* function as core components of the VLCA synthesis complex [23]. Mutations such as cer2 impair the production of wax components longer than 28 carbons, while cer26 affects those longer than 30 carbons [24]. The *CER22* gene, an allele of *CER1*, is also involved in alkane biosynthesis in Arabidopsis [22]. Furthermore, *CsCER1* significantly influences VLCA biosynthesis, cuticle permeability, and drought resistance [25].

In rice, *CER1* plays a crucial role in the biosynthesis of VLCAs [22,26]. The overexpression of *BnCER1-2* in rapeseed not only enhances total alkane deposition but also confers improved drought tolerance phenotypes [27]. Similarly, *PpCER1* in Poa pratensis participates in wax alkane synthesis and contributes to plant drought resistance [28]. In tomato, *SlCER1-1* coordinates alkane biosynthesis, a pathway important for both drought adaptation and postharvest fruit quality [29]. Although these studies collectively establish *CER1* orthologs as key regulators of cuticular wax formation and abiotic stress responses in angiosperms, the specific functions of *CER1* homologs in major monocot crops such as maize—particularly their potential role in modulating antioxidant defense systems—remain poorly understood.

In this study, we investigated the role of *ZmCER1* in maize abiotic stress responses. We first analyzed the expression pattern of *ZmCER1* under drought and salt stress and determined the ZmCER1 subcellular localization. We then validated its function through overexpressing it in both *Arabidopsis thaliana* and maize, evaluating the phenotypic and physiological changes associated with salt and drought tolerance. Finally, we screened for ZmCER1-interacting proteins to explore its molecular mechanism. Combining the physiological indicator data and the results of a yeast two-hybrid screening (Y2H screening) library, we hypothesized that *ZmCER1* enhances abiotic tolerance not only by bolstering the physical cuticular wax barrier but also by regulating ROS scavenging through interactions with peroxisomal proteins. The findings of this study provide novel insights into maize stress adaptation mechanisms and identify candidate genes for genetic improvement of stress-tolerant crops.

## 2. Materials and Methods

### 2.1. Plant Material

The *Z. mays* materials used in this study include the inbred line B104, as well as *ZmCER1* overexpression transgenic lines constructed using B104 as the recipient material. The *Arabidopsis thaliana* materials used were the wild type (WT) Col-0 and *ZmCER1* overexpression lines. In addition, *Nicotiana benthamiana* was also used as experimental material in this study.

### 2.2. Generation of Transgenic Plants

#### 2.2.1. Transgenic *Arabidopsis thaliana* Lines

The target fragment was ligated into the super1300-GFP plant overexpression vector using a double digestion method with Hind III and Spe I restriction enzymes. The recombinant vector was then introduced into *Escherichia coli* DH5α. The plasmid was subsequently transformed into *Agrobacterium tumefaciens* GV3101. The inflorescences of *Arabidopsis thaliana* were immersed in the infection solution and cultured until seeds were harvested. The T0 generation *Arabidopsis thaliana* seeds were sterilized and spotted onto 1/2 MS solid medium containing hygromycin for cultivation. Hygromycin screening and PCR assays were performed to retain positive plants. The positive plants were self-pollinated for two generations, and the homozygous lines were selected through PCR verification and segregation ratio analysis.

#### 2.2.2. Transgenic Maize Lines

Total RNA was extracted from maize leaves using TRIzol reagent (TaKaRa, Dalian, China) and used as a template for cDNA synthesis using the RT reagent kit (TaKaRa, Dalian, China) according to the manufacturer’s protocol. The complete open reading frame (ORF) of maize *ZmCER1* (Gene ID: Zm00001d014055) from the NCBI (http://www.ncbi.nlm.nih.gov/ (accessed on 1 September 2022)) was amplified by PCR with gene specific primers (Table S1), and using the maize leaf cDNA as template, the PCR products were inserted into a pGEM T-easy vector and sequenced. The *ZmCER1* coding sequence was cloned into the PBCXUN vector and transformed into the Agrobacterium tumefaciens strain EHA105 to infect the immature embryo of the inbred line B104 (wild type) to generate transgenic seedlings. PCR-positive plants (T0) were transplanted and self-pollinated for three generations to obtain homozygous transgenic lines.

### 2.3. Gene Expression Analysis

Maize seeds were surface sterilized with 70% (*v*/*v*) ethanol and 0.1% (*w*/*v*) HgCl_2_, rinsed with sterile water, and germinated at 28 °C in darkness for 3 days, then transferred into Hoagland nutrient solution. When the seedlings reached the three-leaf stage, they were treated with a nutrient solution containing 20% polyethylene glycol (PEG), and leaves were collected at 0 h, 4 h, 12 h, 24 h, and 48 h after treatment to analyze gene expression levels by real-time PCR. For salt treatment in soil, maize seeds were sown in pots, and ten days later, uniformly growing seedlings were retained in each pot. An aliquot of 200 mL of 200 mM NaCl solution was watered every two days. Leaves were collected after 1 day, 3 days, 7 days and 10 days of treatment to analyze gene expression levels by real-time PCR. For drought treatment in soil, maize seeds were sown in pots, and ten days later, uniformly growing seedlings were retained in each pot. Then, irrigation of the maize plants was ceased. Leaves were collected after 3 days, 5 days, 7 days and 10 days of treatment to analyze gene expression levels by real-time PCR.

Total RNA was extracted using TRIzol reagent (TaKaRa, Dalian, China) and treated with RNase-free DNase. The total RNA was used as a template for cDNA synthesis using the RT reagent kit (TaKaRa, Dalian, China) according to the manufacturer’s protocol. Real-time PCR was performed on a LightCycler 480 (Roche) with SYBR Green RT-PCR Kit (TaKaRa, Dalian, China) according to the manufacturer’s protocol. Gene expression levels were normalized with the reference gene *Actin1* (GRMZM2G126010) and evaluated using the 2^−△△Ct^ method [30]. The gene specific primers are shown in Table S1.

### 2.4. Subcellular Localization of ZmCER1

ZmCER1 was linked to the green fluorescent protein expression vector 35S-eGFP using the Gateway method. The Gateway BP Cloning Kit (Baiao, Beijing, China) was employed to insert the target gene fragment into the pDONR221 vector. The Gateway LR Cloning Kit (Baiao, Beijing, China) was used to transfer the target fragment into the 35S-eGFP vector. The construct was then introduced into Agrobacterium tumefaciens GV3101 competent cells. The resuspended bacterial solution was injected into tobacco leaves. The leaves were then examined under a laser scanning confocal microscope (Leica, Wetzlar, Germany). *ZmCER1* was cloned into the pBI221-GFP vector. Transient expression of green fluorescent protein (GFP)-fused proteins in maize protoplasts was performed. The fluorescence signal was observed using a confocal laser scanning microscope.

### 2.5. Plant Culture and Treatment

#### 2.5.1. *Arabidopsis thaliana* Culture and Treatment

Sterilized Arabidopsis seeds were sown on 1/2 MS solid medium and vernalized at 4 °C in darkness for 2 days. Afterwards, seeds were moved to a growth chamber and cultured for another 3 days. Seedlings with uniform growth performance were selected and transferred to different stress media for 7 days of treatment.

For drought stress simulation, seedlings were cultivated on 1/2 MS medium supplemented with either 10% PEG 6000 or 200 mM mannitol, both of which function by establishing extracellular osmotic imbalance to mimic drought-induced physiological alterations in plants.

For salt stress treatment, seedlings were grown on 1/2 MS solid medium containing 150 mM NaCl. At the end of the treatment period, relevant physiological and biochemical parameters were measured and analyzed.

For salt stress treatment in soil culture, after 3 days, Arabidopsis plants were transferred into pots, and 7 days later, uniformly growing seedlings were retained in each pot. An aliquot of 200 mL of 200 mM NaCl solution was watered every two days. After 10 days of salt treatment, relevant physiological and biochemical indicators were measured.

For drought stress treatment in soil culture, after 3 days, Arabidopsis plants were transferred into pots, and 7 days later, uniformly growing seedlings were retained in each pot. The plants were subjected to a 10-day drought stress period. Then, the relevant physiological and biochemical indicators are measured.

The Arabidopsis plants were grown at 21–24 °C /18–20 °C (day/night) with a photoperiod cycle of 16 h of light (150–200 μmol m^−2^ s^−1^) at approximately 60–70% relative humidity.

#### 2.5.2. Maize Plant Culture and Treatment

For salt stress treatment, maize seeds were sown in pots, and ten days later, uniformly growing seedlings were retained in each pot. An aliquot of 200 mL of 200 mM NaCl solution was watered every two days. After 10 days of salt treatment, relevant physiological and biochemical indicators were measured.

For drought stress treatment, maize seeds were sown in pots, and ten days later, uniformly growing seedlings were retained in each pot. The plants were subjected to a 10-day drought stress period. Then, the relevant physiological and biochemical indicators were measured.

The maize plants were grown at 25–30 °C/20–25 °C (day/night) with a photoperiod cycle of 14 h of light (500–600 μmol m^−2^ s^−1^) at approximately 65% relative humidity.

### 2.6. H_2_O_2_ Quantification

H_2_O_2_ content was quantified following the method described by Park et al. [31]. Briefly, leaf samples (0.1 g) were homogenized at 4 °C in 5 mL of 50 mM phosphate buffer (pH 6.5) and centrifuged at 10,000× *g* for 20 min. A 3 mL aliquot of the supernatant was then mixed with 1 mL of 0.1% TiCl4 in 20% (*v*/*v*) H_2_SO_4_, followed by centrifugation at 8000× *g* for 5 min. Absorbance was measured at 415 nm to determine H_2_O_2_ concentration using a visible spectrophotometer (Jinghua, Shanghai, China).

### 2.7. MDA Levels

For MDA content determination, the protocol by Peever and Higgins [32] was followed. Absorbance readings of the supernatant were taken at 450, 532, and 600 nm using a visible spectrophotometer (Jinghua, Shanghai, China), and MDA concentration was then calculated using the formula: content (μmol/L) = 6.45 × (A532 − A600) − 0.56 × A450.

### 2.8. Enzyme Determinations

For antioxidant enzyme activity assays, leaf tissues (0.3 g) were homogenized in 8 mL of 50 mM phosphate-buffered saline (pH 7.8) and centrifuged at 10,000× *g* for 15 min at 4 °C. The resulting supernatant was used for subsequent enzymatic analyses. SOD activity was measured following the method of Giannopolitis and Ries [33], while CAT activity was determined according to Alia et al. [34,35].

### 2.9. Relative Water Content (RWC)

The fresh weights (FWs) of the fourth leaves from the base of maize plants were measured. The leaves were then soaked in distilled water for 24 h at room temperature. After soaking, the leaves were blotted dry with filter paper so that the turgid weights (TWs) could be determined. The dry weights (DWs) were obtained after oven-drying for 72 h at 80 °C. The RWC was calculated as follows:

RWC (%) = (FW − DW)/(TW − DW) × 100

### 2.10. Analysis of Wax Composition

Wax extraction and gas chromatography–mass spectrometry (GC–MS) analyses were performed according to the described methods with some modifications [36]. The derivatized samples were analyzed by GC–MS (Agilent gas chromatograph coupled to an Agilent 5973N quadrupole mass selective detector) (Agilent Technologies Inc., Santa Clara, CA, USA).

### 2.11. Yeast Two-Hybrid Screening (Y2H Screening) Assays

The full length of the coding sequence region of *ZmCER1* was inserted into the pBT3–STE vector as a bait that was then transformed into the yeast strain NMY51. The transformed NMY51 yeast strain was then transformed with the maize membrane system yeast cDNA library and placed on the synthetic dropout (SD) medium -Trp-Leu-Ade. Positive clones were selected for sequencing. Then, the selected potential candidate genes were subjected to further point-to-point Y2H assay. Autoactivation was tested by co-transforming bait and prey constructs with their reciprocal empty vector.

### 2.12. Luciferase (Luc) Assay

Full length *ZmCER1* and the promoter fragments of *ZmPEX14* were inserted into the 35S:GFP and pGreenII 0800:LUC vectors, respectively. The recombinant vectors were transformed into *N. benthamiana* leaves using the agrobacterial infiltration method (*A. tumefaciens* strain GV3101) [19]. The luciferase signal was detected using a Tanon 5200 multi-chemiluminescence imaging system (Tanon, Shanghai, China).

### 2.13. Bimolecular Fluorescence Complementation (BiFC) Assay

The coding sequence of *ZmCER1* without stop codon was fused with EYFP on the vector P2YC, while *ZmPEX14* was fused with EYFP on vector P2YN. *Agrobacterium tumefaciens* containing ZmCER1-cYFP and nYFP-ZmPEX14 were used to co-transform the leaves of 1-month-old *N. benthamiana*. Fluorescence of YFP was observed and photographed under a confocal laser scanning microscope (Leica, Wetzlar, Germany), using excitation/emission wavelength 510 nm.

### 2.14. Statistical Analysis

Statistical analysis was performed using SPSS Statistics 23 software (IBM SPSS Statistics Version 23.0). Data were expressed as means ± SD (standard deviation). All experiments were repeated three times, all data were analyzed using Duncan’s multiple-range test and statistical significances were considered at the *p* < 0.05 level.

## 3. Results

### 3.1. Expression Pattern Analysis and Subcellular Localization Analysis

**Figure 1:**
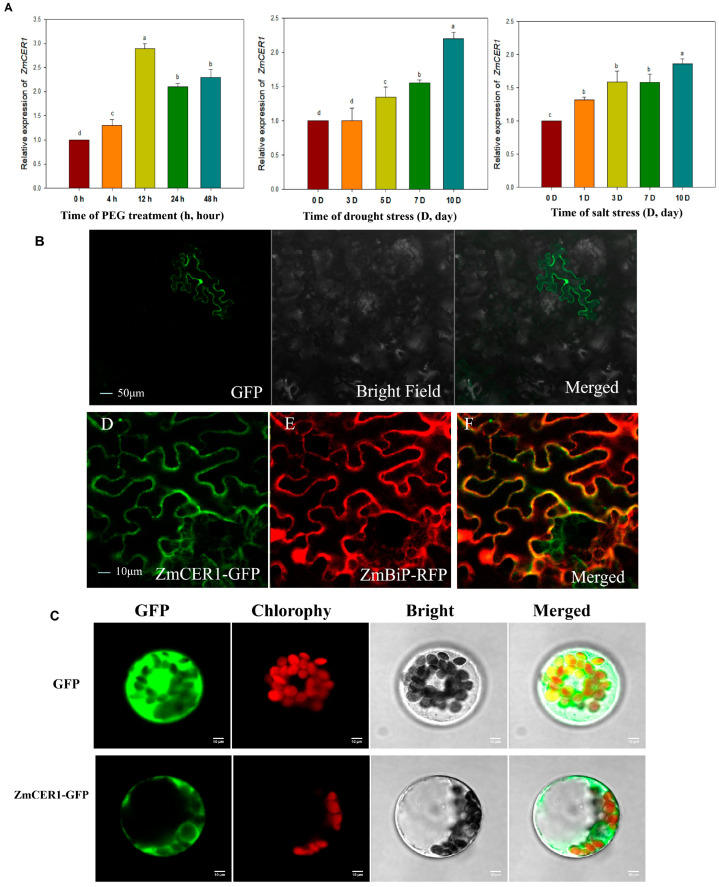
Expression pattern analysis and subcellular localization analysis. (**A**) Relative expression level of *ZmCER1* responsive to PEG, drought stress and salt stress. The expression levels of *ZmCER1* were normalized to that of maize *Actin1*. Different lowercase letters indicate the statistically significant differences between *Arabidopsis thaliana* plants at the *p* < 0.05 level using Duncan’s multiple-range test. Values are means ± SD of three biological replicates. (**B**) Subcellular localization of ZmCER1 in tobacco leaves. ZmBiP, endoplasmic reticulum localization marker. GFP, green fluorescent protein. RFP, red fluorescent protein. (**C**) Subcellular localization of ZmCER1 in maize protoplasts cells. Scale bar = 10 μm.

As shown in Figure 1A, the expression of *ZmCER1* was obviously induced by drought and salt stress, which implied its potential involvement in maize abiotic adaptive responses. The subcellular localization of ZmCER1 was analyzed in *Nicotiana benthamiana* leaves and maize protoplasts cells (Figure 1B,C). In *Nicotiana benthamiana* leaves, the microscopic images showed that the ZmCER1-GFP fluorescence was observed in the endoplasmic reticulum (ER), while the fluorescence of GFP or the ER localization marker control was observed throughout cell or in the ER, respectively. In the maize protoplast cells, the ZmCER1-GFP fluorescence was also observed in the ER.

### 3.2. ZmCER1 Positively Modulates Salt and Drought Tolerance in Arabidopsis thaliana

**Figure 2:**
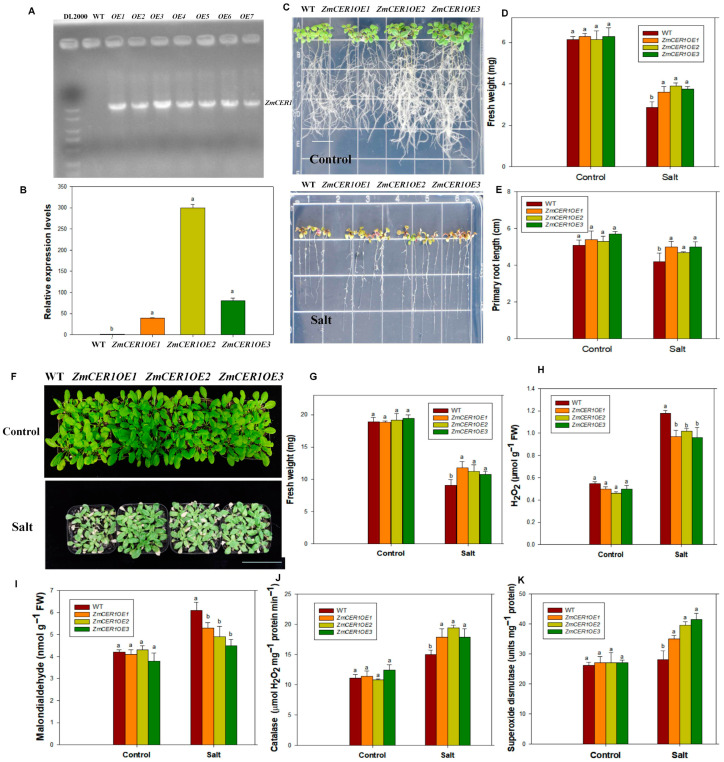
*ZmCER1* positively modulates salt tolerance in *Arabidopsis thaliana.* (**A**) Polymerase chain reaction (PCR) analysis of wild type and transgenic *Arabidopsis thaliana* lines with specific primers for *ZmCER1*. WT, wild type. (**B**) Relative expression levels of ZmCER1 in transgenic *Arabidopsis thaliana* lines. The expression levels of *ZmCER1* were normalized to that of *AtACT2*. (**C**) Phenotypes of *ZmCER1OE* and WT *Arabidopsis thaliana* lines under salt stress on 1/2 MS medium. Scale bar indicates 1 cm. (**D**) Fresh weight. (**E**) Primary root length. (**F**) Phenotypes of *ZmCER1OE* and WT *Arabidopsis thaliana* lines under salt stress in soil. Scale bar indicates 15 cm. (**G**) Fresh weight. (**H**) Hydrogen peroxide (H_2_O_2_) content in leaves. (**I**) Malondialdehyde (MDA) contents in leaves. (**J**) Catalase (CAT) activity in leaves. (**K**) Superoxide dismutases (SOD) activity in leaves. Different lowercase letters indicate the statistically significant differences between *Arabidopsis thaliana* plants under same conditions at the *p* < 0.05 level using Duncan’s multiple-range test. Values are means ± SD of three biological replicates.

In order to investigate the role of *ZmCER1* in regulating abiotic stress tolerance, transgenic *Arabidopsis thaliana* plants that overexpressed *ZmCER1* were generated. A molecular analysis of the transgenic plants was performed. The results indicated the presence of *ZmCER1* and the enhancement in the expression levels of *ZmCER1* in the transgenic plants (Figure 2A,B). Three independent *ZmCER1OE Arabidopsis thaliana* lines were chosen to investigate the role of *ZmCER1* in detail.

Cultured on medium for 7 days for salt tolerance analysis, the growth status of the WT and overexpressing lines remained comparable under normal conditions. However, under NaCl treatments, the overexpressing lines exhibited superior shoot and root growth compared to the WT (Figure 2C). The root length and fresh weight of the *ZmCER1OE* plants were significantly higher than those of the WT (Figure 2D,E). Cultured in soil for further salt tolerance analysis, the *Arabidopsis* plants were treated with 200 mM NaCl for 10 days, and growth retardation was observed in the stress groups relative to the controls, while the overexpressing lines maintained better growth performance than the WT (Figure 2F). The fresh weights of the overexpressing plants were significantly higher than the WT (Figure 2G). The MDA content and the H_2_O_2_ content in the *ZmCER1OE* plants was significantly lower than that in the WT, while the SOD and CAT activities were significantly higher (Figure 2H–K). These results indicate that *ZmCER1* positively modulates salt tolerance in *Arabidopsis thaliana*.

**Figure 3:**
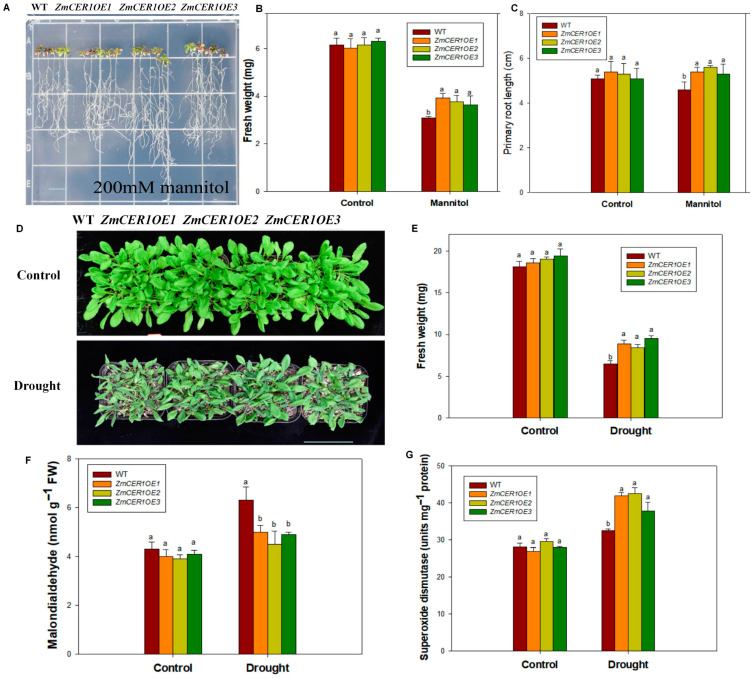
*ZmCER1* positively regulated drought tolerance in *Arabidopsis thaliana*. (**A**) Phenotypes of *ZmCER1OE* and WT *Arabidopsis thaliana* lines under mannitol treatment on 1/2 MS medium. Scale bar indicates 1 cm. (**B**) Fresh weight on medium. (**C**) Primary root length. (**D**) Phenotypes of *ZmCER1OE* and WT *Arabidopsis thaliana* lines under drought stress in soil. Scale bar indicates 15 cm. (**E**) Fresh weight. (**F**) MDA content in leaves. (**G**) SOD activity in leaves. Different lowercase letters indicate the statistically significant differences between *Arabidopsis thaliana* under same conditions at the *p* < 0.05 level using Duncan’s multiple-range test. Values are means ± SD of three biological replicates.

The Arabidopsis plants cultured on 1/2 MS medium for 5 days were then transferred to 1/2 MS medium containing 200 mM mannitol for 7 days. Under osmotic stress conditions, the overexpression lines exhibited superior growth compared to the WT (Figure 3A), with significantly greater fresh weight and root length (Figure 3B,C). Under drought stress in soil for 10 days, the overexpression lines showed better growth than the WT (Figure 3D). Compared to the WT, the treated overexpression lines showed significantly higher biomass and SOD activities and a lower content of MDA (Figure 3E–G). These results indicate that *ZmCER1* positively regulates drought tolerance in *Arabidopsis thaliana*.

### 3.3. ZmCER1 Positively Modulates Salt Tolerance and Drought Tolerance in Maize

**Figure 4:**
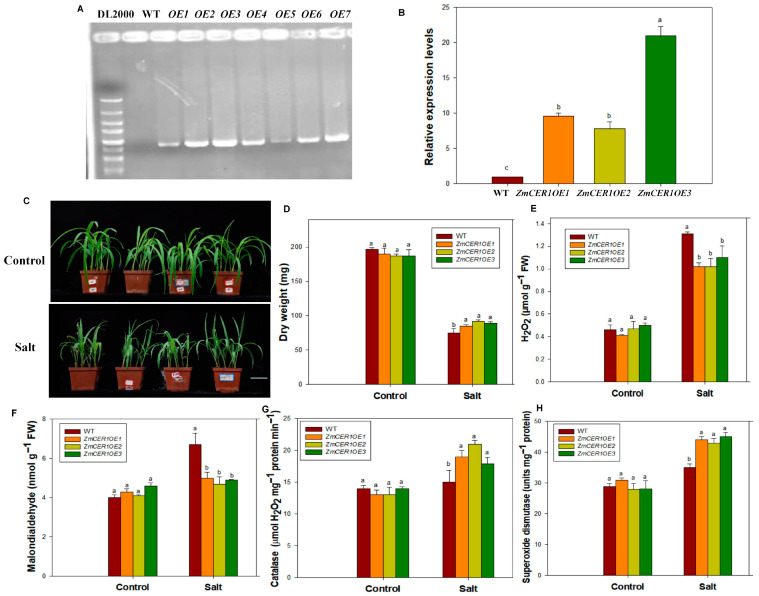
*ZmCER1* positively regulated salt tolerance in maize. (**A**) PCR analysis of wild type and transgenic maize with specific primers for *ZmCER1*. WT, wild type. (**B**) Relative expression levels of *ZmCER1* in transgenic maize lines. The expression levels of *ZmCER1* were normalized to that of maize *Actin1*. The wild type (WT) was set as the reference control. Different lowercase letters indicate the statistically significant differences between maize lines at the *p* < 0.05 level using Duncan’s multiple-range test. Values are means ± SD of three biological replicates. (**C**) Phenotypes of *ZmCER1OE* and WT maize plants under salt stress in soil. Scale bar indicates 10 cm. (**D**) Dry weight. (**E**) H_2_O_2_ content in leaves. (**F**) MDA content in leaves. (**G**) CAT activity in leaves. (**H**) SOD activity in leaves. Different lowercase letters indicate the statistically significant differences between maize plants under same conditions at the *p* < 0.05 level using Duncan’s multiple-range test. Values are means ± SD of three biological replicates.

Transgenic maize plants that overexpressed *ZmCER1* were generated. A molecular analysis of the transgenic maize plants was performed. The results indicated the presence of *ZmCER1* and an enhancement in the expression levels of *ZmCER1* in the transgenic maize plants (Figure 4A,B). Three independent *ZmCER1OE Arabidopsis thaliana* lines were chosen to investigate the role of *ZmCER1* in detail.

After the maize plants reached the three-leaf stage, they were treated with 200 mM NaCl for 10 days. Under normal conditions, all the lines exhibited relatively uniform growth. Salt stress exposure significantly increased adverse effects on growth across all the lines. The WT plants exhibited more pronounced wilting and desiccation than the *ZmCER1OE* maize plants (Figure 4C), indicating that the overexpression of *ZmCER1* enhances salt stress tolerance in maize. Concurrently, various physiological parameters supported this conclusion. Under salt stress, the dry weight and activity of CAT and SOD were significantly higher in the *ZmCER1OE* maize lines, while the MDA and H_2_O_2_ content was lower than that in the WT (Figure 4D–H). This suggests that the *ZmCER1* overexpression lines might confer enhanced salt tolerance through an improved ROS-scavenging capacity.

**Figure 5:**
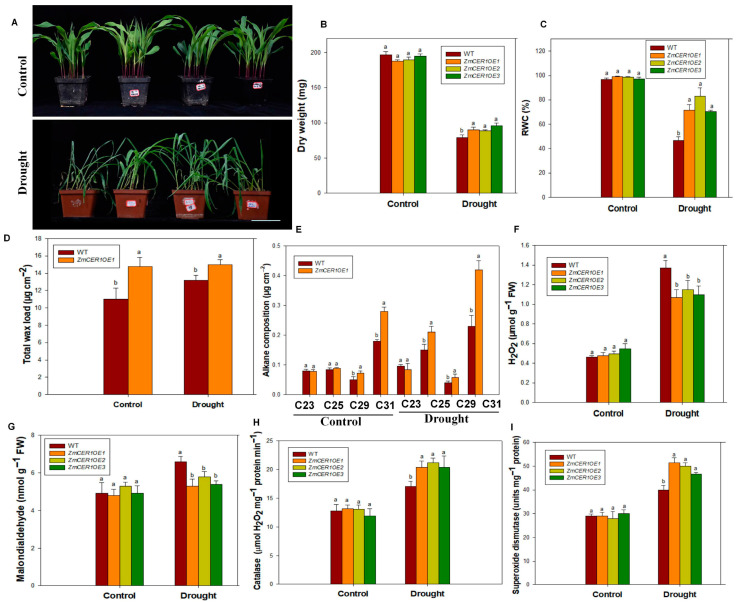
*ZmCER1* positively regulated drought tolerance in maize. (**A**) Phenotypes of *ZmCER1OE* and WT maize plants under drought stress. Scale bar indicates 10 cm. (**B**) Dry weight. (**C**) Relative water content (RWC) in leaves. (**D**) Total wax loads on maize leaf surfaces. (**E**) Amount of alkanes on maize leaf surfaces. (**F**) H_2_O_2_ content in leaves. (**G**) MDA contents in leaves. (**H**) CAT activity in leaves. (**I**) SOD activity in leaves. Different lowercase letters indicate the statistically significant differences between maize plants under same conditions at the *p* < 0.05 level using Duncan’s multiple-range test. Values are means ± SD of three biological replicates.

Drought treatment was initiated after the maize reached the three-leaf stage. Under normal conditions, the growth of all the lines was relatively uniform. Under drought treatment for 10 days, the growth of all the plants was significantly impaired. The WT exhibited more severe leaf wilting and plant lodging compared to the *ZmCER1OE* plants (Figure 5A), indicating that the overexpression of *ZmCER1* enhances maize tolerance to drought stress. Concurrently, various physiological indices supported this conclusion. Under drought stress, the dry weight and WRC were significantly higher in the *ZmCER1OE* lines than those in the WT (Figure 5B,C). Additionally, the amount of total wax and alkane content—especially that of C29 and C31—were significantly increased in the *ZmCER1OE* lines compared to the WT (Figure 5D,E). Moreover, the CAT and SOD activities were significantly higher in the *ZmCER1OE* maize lines, while the MDA and H_2_O_2_ contents were lower than that in the WT (Figure 5F–I). These results suggest that the overexpression of *ZmCER1* might confer enhanced drought tolerance in maize through the improved biosynthesis of cuticular waxes and ROS-scavenging capacity.

### 3.4. Screening of Interacting Proteins for ZmCER1

**Figure 6:**
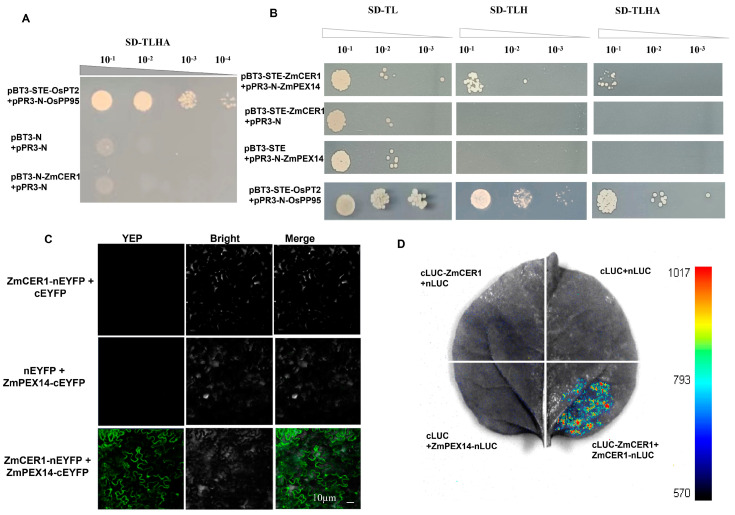
Interaction analysis of ZmCER1 with ZmPEX14. (**A**) Analysis of the self-activation of ZmCER1. (**B**) Yeast two-hybrid (Y2H) assay. OsPT2, rice phosphate transporter 2. OsPP95, rice purple acid phosphatase 95. (**C**) Bimolecular fluorescence complementation (BiFC). (**D**) Firefly luciferase complementation imaging (LUC).

To elucidate the mechanisms by which *ZmCER1* confers stress resistance in maize, we employed a Y2H assay to screen for proteins interacting with ZmCER1. First, we tested whether ZmCER1 exhibits autoactivation activity. It was observed that yeast cells carrying the ZmCER1 recombinant vector failed to grow on SD/-Leu/-His/-Trp/-Ade medium (Figure 6A), indicating that the ZmCER1 protein does not exhibit autoactivation activity in yeast, confirming its suitability for subsequent Y2H screening. The Y2H screen was performed to identify proteins interacting with ZmCER1. A total of 123 yeast colonies were obtained, followed by a PCR amplification of these colonies. A total of 15 candidate genes were obtained (Table S2). Among them, the peroxisomal membrane protein ZmPEX14 was selected for further interaction analysis. The results of the Y2H assay showed that ZmPEX14 possibly interacts with ZmCER1 (Figure 6B). The interactions were further confirmed by BiFC and LUC (Figure 6C,D).

### 3.5. ZmPEX14 Positively Modulates Salt Tolerance and Drought Tolerance in Arabidopsis

**Figure 7:**
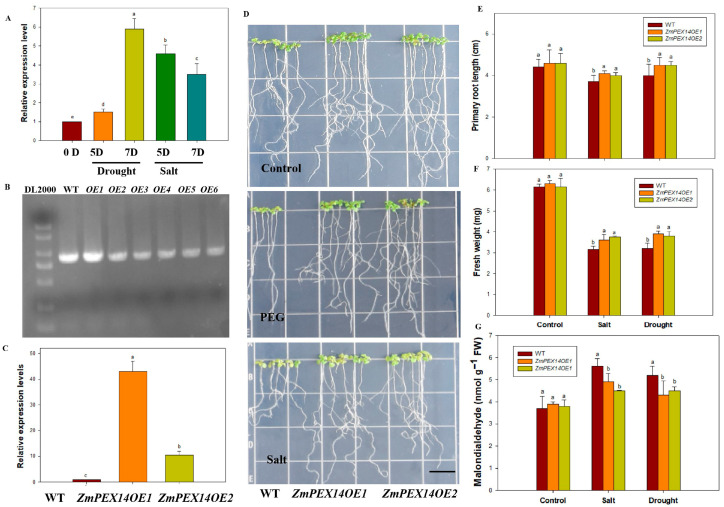
*ZmPEX14* positively modulates drought and salt tolerance in *Arabidopsis thaliana.* (**A**) Relative expression levels of *ZmPEX14* responsive to drought and salt stress. The expression levels of *ZmPEX14* were normalized to that of maize *Actin1*. Different lowercase letters indicate the statistically significant differences between *Arabidopsis thaliana* plants at the *p* < 0.05 level using Duncan’s multiple-range test. Values are means ± SD of three biological replicates. (**B**) PCR analysis of wild type and transgenic *Arabidopsis thaliana* lines with specific primers for *ZmPEX14*. WT, wild type. (**C**) Relative expression levels of *ZmPEX14* in transgenic *Arabidopsis thaliana* lines. The expression levels of *ZmPEX14* were normalized to that of *AtACT2*. (**D**) Phenotypes of *ZmPEX141OE Arabidopsis thaliana* lines and WT under PEG 6000 (10%) and salt stress on 1/2 MS medium. Scale bar indicates 1 cm. (**E**) Primary root length. (**F**) Fresh weight. (**G**) MDA contents in leaves. Different lowercase letters indicate the statistically significant differences between *Arabidopsis thaliana* plants under same conditions at the *p* < 0.05 level using Duncan’s multiple-range test. Values are means ± SD of three biological replicates.

As shown in Figure 7A, the expression of *ZmPEX14* was obviously induced by drought and salt stress, which implied its potential involvement in abiotic adaptive responses. The transgenic *Arabidopsis* plants that overexpressed *ZmPEX14* were generated to analyze the role of *ZmPEX14* in drought and salt tolerance. The results of the molecular analysis indicated the presence of the *ZmPEX14* and the enhancement in the expression levels of *ZmPEX14* in transgenic *Arabidopsis* plants (Figure 7B,C). As shown in Figure 7D, when cultured on a medium supplemented with 5% PEG or 100 mM NaCl for 5 days, the *ZmPEX14OE* lines showed better growth status than the WT, and the root length and fresh weight of the *ZmPEX14OE* plants were significantly higher than those of the WT, while the MDA content was lower than that in the WT (Figure 7E–G). Given that the peroxisome has been reported to be associated with the scavenging of reactive oxygen species in plants, which is crucial for abiotic stress tolerance [37,38], these findings suggest that *ZmCER1* may regulate maize abiotic stress tolerance by influencing the scavenging of ROS through the interaction with ZmPEX14.

## 4. Discussion

The escalating threat of abiotic stresses, particularly drought and salinity, poses a significant challenge to global food security, with maize (*Zea mays* L.) being exceptionally vulnerable [39,40]. The plant cuticle, a hydrophobic barrier composed of cutin and cuticular waxes, serves as the first line of defense against environmental adversities [10,12]. Within the cuticular waxes, VLCAs are critical components that significantly reduce epidermal permeability [17]. The enzyme aldehyde decarbonylase, encoded by the CER1 gene in Arabidopsis, is a key catalyst in the alkane-forming pathway [21]. Although many studies collectively establish *CER1* orthologs as key regulators of cuticular wax formation and abiotic stress responses in angiosperms, the specific functions of *CER1* homologs in major monocot crops such as maize—particularly their potential role in modulating antioxidant defense systems—remain poorly understood. In this study, we functionally characterized *ZmCER1*, demonstrating its pivotal role in VLCA biosynthesis, its ROS-scavenging ability and its positive regulation of drought and salt tolerance in both model and crop plants.

The induction of *ZmCER1* expression by drought and salt stress (Figure 1A) is a hallmark of stress-responsive genes involved in stress adaptive responses. This pattern aligns with observations in other species, for instance, *BnCER1-2* in Brassica napus and *PpCER1* in Poa pratensis are similarly upregulated by abiotic stress and their overexpression enhances drought tolerance [27,41]. The subcellular localization of ZmCER1 to the ER (Figure 1B,C) is consistent with the established understanding of cuticular wax biosynthesis, where the ER serves as the primary site for the synthesis of VLCFA precursors [11,15]. The most compelling evidence for *ZmCER1*’s function comes from the phenotypic analysis of the overexpression lines. The heterologous overexpression of *ZmCER1* in *Arabidopsis thaliana* conferred enhanced tolerance to both salt and drought stresses (Figure 2 and Figure 3). Crucially, we validated these findings in maize. The overexpression of *ZmCER1* in maize led to a marked improvement in salt and drought tolerance (Figure 4 and Figure 5). The consistent positive effects in both *Arabidopsis* and maize underscore its potential as a prime target for molecular breeding.

Beyond the morphological advantages, the physiological data point to a robust mechanism underlying the stress tolerance conferred by *ZmCER1*. In both *Arabidopsis* and maize overexpression lines, we observed a consistent pattern: under stress conditions, the overexpression lines possessed significantly higher activities of the antioxidant enzymes CAT and SOD, coupled with lower levels of H_2_O_2_ and MDA (Figure 4 and Figure 5). MDA is a key marker of lipid peroxidation, and its accumulation indicates severe oxidative damage to cell membranes [9]. The reduced MDA and ROS levels in the *ZmCER1*-overexpressing maize plants suggest that the cellular membranes are better protected against oxidative damage under salt and drought stress. This finding strongly suggests that *ZmCER1* enhances stress tolerance not merely by reinforcing the physical cuticular barrier but also by boosting the cellular ROS-scavenging capacity.

An intriguing question emerged regarding the influence of alkane synthase on the antioxidant system. Our protein interaction screening has now provided a key molecular link. The identification of ZmPEX14, a peroxisomal membrane protein, as a potential interacting partner of ZmCER1 is a finding of significant interest (Figure 6, Table S2). Peroxisomes in plants contain approximately 200 types of proteins and participate in various primary and secondary metabolic pathways, including photorespiration, fatty acid degradation, phytohormone biosynthesis, and ROS detoxification, and play crucial roles in plant growth, development, and stress responses while significantly influencing crop yield and stress resistance [42,43]. Moreover, peroxisomes contain various ROS-scavenging proteins, including CAT and SOD [44,45].

**Figure 8:**
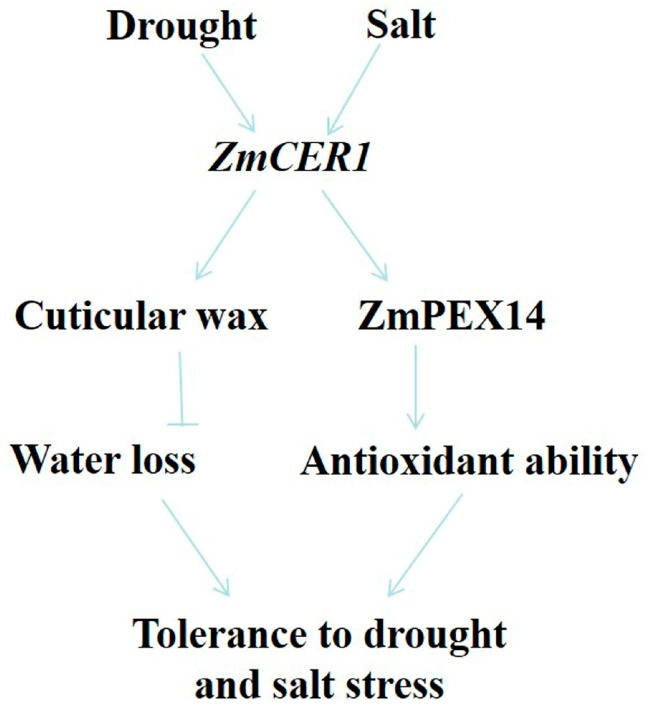
A proposed model for the role of *ZmCER1* in regulating drought and salt tolerance. *ZmCER1* functions not only by bolstering the physical cuticular wax barrier but also by potentiating the cellular antioxidant system via an interaction with the peroxisomal protein ZmPEX14. Arrows indicate positive regulation, bold blunt-ended bars indicate inhibition.

The proteins involved in peroxisome assembly are primarily peroxins (PEX), including the membrane proteins PEX3, PEX16, PEX19, PEX13, PEX14, PEX22, and PEX26; the receptor proteins PEX5 and PEX7; the E2 ubiquitin-conjugating enzyme PEX4; the E3 ubiquitin ligases PEX2, PEX10, and PEX12; and the two AAA ATPases PEX1 and PEX6, among others. These PEX proteins are highly conserved across eukaryotes. Defects in these proteins impair proper peroxisome formation, affecting their number, morphology, and overall functional integrity [46]. Studies have shown that this class of proteins is involved in plant stress response processes. For instance, under H_2_O_2_-induced oxidative stress, the expression of AtPEX1, AtPEX5, and AtPEX14 is up-regulated [47]. Under salt stress, the expression of AtPEX10 and AtPEX1 is induced [48]. Under drought stress, AtPEX14 is positively regulated to enhance drought tolerance in Arabidopsis [49]. Under cadmium stress, AtPEX7 is essential for the correct import of peroxisomal matrix proteins and plays a critical role in the Arabidopsis root response to cadmium stress and in scavenging ROS accumulation [50]. In this study, we found that the overexpression of *ZmPEX14* obviously improved drought and salt stress in *Arabidopsis* (Figure 7). Thus, we propose a dual-mode model for *ZmCER1*-mediated stress tolerance (Figure 8). First, as a direct effector, *ZmCER1* enhances the synthesis of VLCFA, fortifying the cuticular layer and reducing non-stomatal water loss, the primary benefit of a robust cuticle [18]. Second, as a signaling or regulatory node, ZmCER1 may influence peroxisomal activity through its interaction with ZmPEX14. This interaction might potentially modulate the import of antioxidant enzymes into peroxisomes or influence peroxisomal metabolism, thereby enhancing the cell’s overall capacity to detoxify ROS. In conclusion, our study unequivocally identifies *ZmCER1* as a key genetic determinant for improving abiotic stress tolerance in maize. It functions not only by reinforcing the physical cuticular wax barrier but also, we hypothesize, by potentiating the cellular antioxidant system via an interaction with the peroxisomal protein ZmPEX14. This dual role makes *ZmCER1* an exceptionally attractive candidate for precision breeding. Deploying favorable *ZmCER1* alleles or editing its promoter region in elite maize cultivars holds great promise for developing new varieties capable of sustaining yield in the face of climate change-induced water scarcity and soil salinization.

## 5. Conclusions

This study confirms that maize *ZmCER1*, a key gene involved in cuticular wax alkane biosynthesis, serves as a critical regulator of salt and drought tolerance in maize. The core findings demonstrate that *ZmCER1* exerts its protective effects through two interconnected mechanisms: reinforcing the cuticular wax barrier and regulating ROS detoxification.

Functionally, *ZmCER1* is induced by stress and encodes an ER-localized protein. Both heterologous overexpression in Arabidopsis and homologous overexpression in maize significantly enhance the transgenic plants’ stress tolerance, which is associated with elevated activities of antioxidant enzymes (SOD and CAT), reduced levels of MDA and H_2_O_2_, and—specifically in maize—increased contents of cuticular wax alkanes that reduce non-stomatal water loss by strengthening the hydrophobic barrier.

Molecularly, we identified ZmPEX14, a stress-inducible peroxisomal membrane protein, as a direct interacting partner of ZmCER1, which was validated by Y2H, LUC, and BiFC assays. Consistent with its interaction with ZmCER1, the overexpression of *ZmPEX14* also improves stress tolerance in Arabidopsis, suggesting a synergistic role of the *ZmCER1*–*ZmPEX14* complex in modulating ROS detoxification.

Collectively, our findings establish that *ZmCER1* mediates maize stress tolerance by integrating cuticular wax barrier fortification and peroxisomal ROS detoxification via interaction with ZmPEX14. This validates *ZmCER1* as a promising candidate gene for molecular breeding strategies aimed at improving maize stress tolerance and ensuring stable yields under the challenges of climate change.

## Data Availability

Data supporting the findings of this study are available in the article and its Supplementary Materials.

## Supplementary Materials

The following supporting information can be downloaded at: https://www.mdpi.com/article/10.3390/cimb48050509/s1, Table S1: PCR primers used in this study; Table S2: Two-hybrid (Y2H) screen of ZmCER1.

## Abbreviations

The following abbreviations are used in this manuscript:

*ZmCRE1*	Maize aldehyde decarbonylase 1 gene
MS	Murashige and Skoog
RT-PCR	Real-time polymerase chain reaction
ROS	Reactive oxygen species
MDA	Malondialdehyde
RWC	Relative water content
SOD	Superoxide dismutase
CAT	Catalase
VLCFAs	Very-long-chain fatty acids
Y2H	Yeast two-hybrid
LUC	Luciferase complementation
BiFC	Bimolecular fluorescence complementation
