# Supplementary material for: Maize Aldehyde Decarbonylase 1 Gene (ZmCER1) Positively Regulates Salt and Drought Tolerance by Improving Wax Synthesis and Reactive Oxygen Species Detoxification
Source: Curr Issues Mol Biol. 2026 May 14;48(5):509. doi: 10.3390/cimb48050509 (PMC13204785; doi:10.3390/cimb48050509)
Supplement: Supplementary file 1 [file cimb-48-00509-s001.zip › Table S1. PCR primers used in this study.pdf]

Table S1. PCR primers used in this study.

Primer		Sequence 5'-3'	Description
ZmCER1	F	AGCAGTTGCGGGTGTAGC	Used for *ZmCER1* cloning
	R	TGATCTTTGAGCGTAGGG	
ZmCER1	F	TGCCAAGTGGTATGTGCG	Used for *ZmCER1* qRT-PCR
	R	ATTGATTGCTTCCTTCTCCC	
Actin	F	CGAGAAGAGCTACGAGATGC	
	R	CCCACTGAGGACAACGTTAC	
ZmCER1	F	GGGGACAACTTTGTACAAAAAAGTTGGC ATGGCGACGAACCCCGGC	Used for ZmCER1 subcellular localization
	R	GGGGACAACTTTGTACAAGAAAGTTGGGCA AACTTGAGCCACAGGGCGG	
ZmCER1	F	CCCAAGCTTGGGATGGCGACGAACCCCGGCCT	Used for constructing Arabi dopsis overexpres sion vectors
	R	GGACTAGTCCAACTTGAGCCACAGGGCGGAA	
1300-GFP	F	TCAGAAATGGATAAATAGCCTTGC	Used for *ZmCER1* overexpression A rabidopsis lines identification
	R	TGAACTTGTGGCCGTTTACGTC	
pBT3-N	F	CTTGATATCGAATTCCTGCAGATGGCGACGAACCCC GGC	Used for yeast vector construction
	R	GCGGTTAGCTACTTACCATGGAACTTGAGCCACAGG GCGG	
ZmCER1	F	GTCACCGAGCCAATCACC	Used for *ZmCER1* overexpression maize lines identification
	R	GGCAACAGCACGAGCAAC	
Origin	F	ACTAGGGTCTCGCACCATGGCGACGAACCCCGGC	Used for constructing maiz e overexpression vectors
	R	ACTAGGGTCTCTACCG TCAAACTTGAGCCACAGGGCGG	
pPR3-N	F	CGGTAAAACCGGAACATTGGA	Used for identification of interacting genes
	R	ACTTCAGGTTGTCTAACTCCT	
	R	CGGTATCTGAGTCCAGGTCGTC	
ZmPEX14	F	ACCCAGCTCCGCGCCGCAG	Used for *ZmPEX14* cloning
	R	CTATGCAGATGCATATGCATAT	
ZmPEX14	F	GTACTCATGAAGTTAACAGA	Used for *ZmPEX14* qRT-PCR
	R	CGCACACCTAAACAGTGACCA	
ZmPEX14	F	CCCAAGCTTGGGATGGCGAGAAGTCACCCA	Used for constructing Arabi

	R	GGACTAGTCCAACTTCAAGAGGCTTCCTGCTCCTG	dopsis overexpression vectors
ZmPEX14	F	CTGGTAACAAAGATGATGGCA	Used for *ZmPEX14* overexpression Arabidopsis lines identification
	R	TGTCAATAGCTTTGATGGTCC	
